# Lightweight Artificial Aggregates Produced from Water Reservoir Sediment and Industrial Waste—Ecological and Technological Aspect

**DOI:** 10.3390/ma18112563

**Published:** 2025-05-30

**Authors:** Adam Masłoń, Maksymilian Cieśla, Renata Gruca-Rokosz, Lesław Bichajło, Andrzej Nowotnik, Maciej Pytel, Kamil Gancarczyk, Marcin Chutkowski, Marek Potoczek, Małgorzata Franus, Katarzyna Kalinowska-Wichrowska

**Affiliations:** 1 Department of Environmental Engineering and Chemistry, Rzeszow University of Technology, Powstańców Warszawy 12 Av., 35-959 Rzeszów, Poland; cmax@prz.edu.pl (M.C.); renatagr@prz.edu.pl (R.G.-R.); 2 Department of Roads and Bridges, Rzeszow University of Technology, Powstańców Warszawy 12 Av., 35-959 Rzeszów, Poland; leszbich@prz.edu.pl; 3 Department of Material Science, Rzeszow University of Technology, Powstańców Warszawy 12 Av., 35-959 Rzeszow, Poland; nowotnik@prz.edu.pl (A.N.); mpytel@prz.edu.pl (M.P.); kamilgancarczyk@prz.edu.pl (K.G.); 4 Department of Chemical and Process Engineering, Rzeszow University of Technology, Powstańców Warszawy 12 Av., 35-959 Rzeszów, Poland; ichmch@prz.edu.pl; 5 Department of Industrial and Materials Chemistry, Rzeszow University of Technology, Powstańców Warszawy 12 Av., 35-959 Rzeszów, Poland; potoczek@prz.edu.pl; 6 Department of General Construction, Lublin University of Technology, Nadbystrzycka 40 Street, 20-618 Lublin, Poland; m.franus@pollub.pl; 7 Institute of Civil Engineering, Bialystok University of Technology, Wiejska 45E Street, 15-351 Bialystok, Poland; k.kalinowska@pb.edu.pl

**Keywords:** artificial aggregates, bottom sediment, concrete dust, fly ash, recycling

## Abstract

The use of mineral waste for the production of lightweight artificial aggregate is an important element of activities for sustainable development in construction and the implementation of the objectives of the circular economy. The article presents the physical, mechanical, and ecological properties of an innovative artificial aggregate produced from bottom sediments, concrete dust, and municipal solid waste incineration fly ash. The obtained research results confirm that the developed material achieves technological properties comparable to artificial aggregates available on the market, both commercial and those derived from recycling. However, the increased leachability of chlorides and sulphates remains a significant challenge, which may limit the scope of its applications. Despite this, the material shows the potential for use, among others, in the production of lightweight concrete. The analyses carried out have shown that the thermal hardening processes (200–400 °C) and autoclaving do not guarantee full immobilization of harmful substances contained in the raw materials for the production of this type of aggregate.

## 1. Introduction

Current global development is characterized by the growing consumption of natural resources and energy. This development and technological progress have made humans the largest consumers of natural resources and producers of waste. Despite its undeniable benefits, this development brings with it many adverse problems, such as environmental pollution, greenhouse gas emissions, and threats to human health [1]. In order to reduce the amount of waste and its negative impact on the environment, a waste management policy has been introduced—the circular economy [2]. The concept of the circular economy is based on the rational use of resources and the reduction of the negative impact of manufactured products on the environment. This model aims to minimize the consumption of raw materials and the generation of waste, thereby reducing emissions and energy use, by creating a closed loop of processes in which the waste generated is treated as raw material in subsequent production stages [3,4].

Bottom sediments are accumulated sedimentary material that deposits systematically, leading to a reduction in the retention capacity of water basins, especially dam reservoirs. They are a natural element of the aquatic ecosystem, containing both organic and inorganic substances of natural and anthropogenic origin. Excessive accumulation of bottom sediments contributes to a reduction in the capacity and depth of reservoirs, which limits their utility. Due to the rapid rate of suspended solids accumulation, especially in small water bodies, it is necessary to periodically remove them. Reclamation of water bodies, especially through dredging, is a key process for restoring the function of water bodies and improving their ecological condition. Bottom sediments obtained by dredging can be used in agriculture to improve soil structure [5] and for the restoration of degraded areas [6]. Some of these sediments can play the role of natural fertilizers [7], as they are extracted from the bottom of water reservoirs and rivers and are a rich source of organic matter and easily assimilable organic compounds. However, their use for agricultural purposes may be problematic due to high contamination with heavy metals and organic compounds such as pesticides, PAHs, and PCBs [8]. The bottom sediments from dredging can be used, among other things, in road construction [9], in construction as a component of concrete [10], for cement production [11], for mortar production [12], and for geopolymer mortar production [13], for the production of partition blocks and paving blocks [14], cement bricks [15], extruded aggregates [16], and lightweight aggregates [17,18].

The development of construction causes the generation of significant amounts of construction and demolition waste, which is becoming a major environmental problem [19]. Construction and demolition waste (CWD) is the largest waste stream in the European Union. In the EU, over 850 million tons of construction and demolition waste are generated, accounting for 40% of the whole waste mass [20]. In China and India, on the other hand, over 3 trillion tons of this waste are generated [19]. It is estimated that construction and building operation are also responsible for 37% of global CO_2_ emissions. The production and use of materials such as cement, steel, and aluminium have a significant carbon footprint [21]. Waste from construction, renovation, and demolition work is characterized by a high degree of material diversity. The largest material fraction on average in CDW is the mineral fraction (77%). It consists mainly of concrete (24.0%), bricks (5.0%), and tiles and ceramics (1.2%). The remaining share is made up of other inert waste (46.9%). In the non-mineral waste fraction, metals, glass, paper, and plastic can be separated [20]. In the context of sustainable development, the management of construction waste, including concrete rubble, is becoming a key element of environmental protection strategies and the circular economy. The vast majority of construction and demolition waste can be recycled and reused [22]. The analysis of the topic shows that construction rubble can be reused, for example, for the production of mortar [23], geopolymer mortar [24], recycled concrete aggregates [25,26], paving blocks [27], or lightweight artificial aggregates [28,29].

Mass waste incineration is currently one of the most important technologies used in municipal waste management in the most industrialized countries of the world [30]. In some countries, it is even the dominant technology (e.g., Japan, Switzerland), contributing over 70% to waste management. Currently, there are over 2700 waste incineration plants (operating with various technologies) worldwide, with approximately 500 installations in Europe [31]. The residues generated from waste incineration primarily consist of municipal solid waste incineration fly ash (MSWI-FA) and bottom ash (MSWI-BA) [32]. Annual ash production is estimated at over 2 billion tons [33], which therefore requires the use of appropriate treatment and management methods. A review of the literature reveals the availability of a wealth of information on the applications of municipal waste incineration ash [34,35]. MSWI-FA can be used in agriculture [36], in road construction for embankments [37], for the production of asphalt mortar [38], for the production of building materials, including cement [39], cement mortar [40], geopolymer mortar [41], concrete [42], lightweight aggregates [43], glass-ceramics [44], or use as a pollutants adsorbent [45] or in wastewater treatment plants for sewage sludge treatment [46].

A material with a grain density of less than 2000 kg/m^3^ or a bulk density of less than 1200 kg/m^3^ is considered an artificial lightweight aggregate [47]. These properties are mainly due to the presence of closed pores in the particle structure and surface bubbles, which are formed as a result of thermal transformations of the raw material [48]. The hardening process of fresh aggregates can be accomplished through sintering, cold bonding, or autoclaving to achieve the desired strength of the aggregate [49]. Classic lightweight aggregates are produced from mineral raw materials—swelling clays and silts, and these include, among others: expanded clay, expanded shale, and slate aggregate [50]. Analysis of the subject also indicates the possibility of using various types of waste for the production of lightweight aggregates, e.g., fly ash [51], sewage sludge [52], glass [53], and even plastics [54]. Thanks to their properties, lightweight artificial aggregates are widely used in construction and can serve as an alternative to natural aggregates [48]. Intensive research is currently underway on the production of lightweight, environmentally friendly artificial aggregates. Key areas of research include the search for new waste substrates for their production and the development of more environmentally friendly production processes. Ultimately, these activities aim to achieve a balance between production efficiency and costs on the one hand, and to reduce the negative industrial pressure of humans on the environment on the other. Lightweight artificial aggregates are an innovative solution in construction, offering a number of economic and environmental benefits. As research and technology advance, their use is likely to grow, contributing to more sustainable and efficient construction. Moreover, a review of the literature clearly indicates a lack of knowledge in the field of low-temperature and/or autoclaved production of lightweight artificial aggregates. In this context, their study opens up new, and more environmentally friendly, solutions for the implementation of a circular economy.

The main objective of the study was to optimize the composition and parameters of the production process of ecological lightweight aggregates produced from bottom sediments, concrete dust from recycled concrete, and municipal solid waste incineration fly ash. Next, the thesis on the effectiveness and possibility of leaving low-temperature sintering and autoclaving in the production process of lightweight artificial aggregates was verified.

## 2. Materials and Methods

### 2.1. Raw Materials

**Figure 1:**
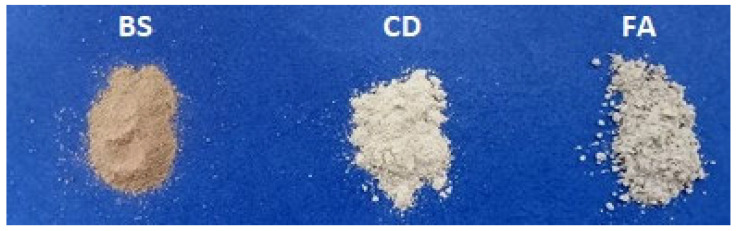
Raw materials used in this study.

In these studies, mineral waste materials were used—bottom sediment (BS), concrete dust (CD), and municipal solid waste incineration fly ash (FA) (Figure 1). Bottom sediment was collected from the Nielisz reservoir (Poland), then dried, ground, and sieved to a fraction of <1 mm. Concrete dust was waste from grinding concrete ceilings. Fly ash came from the Białystok’s Municipal Waste Treatment Plant. Both concrete dust and fly ash had a natural powder form. These materials were not subject to additional mechanical treatment before. The specific density of bottom sediment, concrete dust, and fly ash was 2.56 g/cm^3^, 2.52 g/cm^3^, and 2.57 g/cm^3^, respectively.

### 2.2. Methods of Production of Lightweight Artificial Aggregates

The procedure for producing lightweight aggregate consisted of mixing three mineral raw materials—bottom sediment, concrete dust, and fly ash with the addition of CaO, then granulating, drying, and hardening—sintering or autoclaving. The weight ratio BS:CD:FA was 0.4:0.4:0.2 for all the specimens. CaO was added to the raw material mixture in the amount of 6% by weight. Table 1 presents the organizational plan for the production of lightweight aggregate.

The granulation process took place at a rotational speed of 25–30 rpm and a bowl inclination of 40°. The adopted thermal treatment temperature was determined experimentally and allowed obtaining good strength parameters and density of aggregates, which allowed them to be classified as lightweight in accordance with the BS EN 13055:2016 standard [47]. The developed technology for the production of lightweight aggregate from bottom sediments, concrete dust, and fly ash is the subject of two patent applications P.451564 [55], P.451565 [56] and a research project (see the Funding).

### 2.3. Methods Analitical Raw Materials and Aggregates

Both the raw materials and the lightweight aggregate obtained were subjected to physicochemical and mechanical tests.

The particle size distribution of the raw materials was measured using a Mastersizer 3000 laser particle size analyzer (Malvern Panalytical, Malvern, UK) with a Hydro EV dispersing attachment (Malvern Panalytical, Malvern, UK).

The morphology of the raw materials and the lightweight aggregate was assessed using a Hitachi S-3400N scanning electron microscope (HITACHI, Tokyo, Japan). Macro-up to approx. 50× and microscopic > 50× observations of the samples were performed using a backscattered electron detector (Backscattered Electrons) (Thermo Fisher Scientific, Waltham, MA, USA), at an accelerating voltage of 20–25 kV, high vacuum mode < 1 Pa, and low vacuum mode of approx. 40–50 Pa. The distance of the electron beam focal point from the WD objective lens pole piece was approx. 10 mm. Observations were performed in the magnification range from 8× to 1000×. The content of elements in the tested sintered samples and in the form of powders was determined using the Thermo Scientific™ UltraDry EDS detector (Thermo Fisher Scientific, Waltham, MA, USA) for analysis of chemical composition by X-ray dispersive spectroscopy—Energy Dispersive Spectroscopy (EDS) and Thermo Scientific™ NORAN™ System 7 software (Thermo Fisher Scientific, Waltham, MA, USA).

The mineral composition of the raw materials was determined by the powder method using X-ray diffraction using the Panalytical X’pert APD X-ray diffractometer (Malvern Panalytical, Malvern, UK) with a PW 3020 goniometer and a Cu lamp and a graphite monochromator. Analyses were performed in the angular range of 5–65 2θ. X’Pert software (https://www.malvernpanalytical.com/en/support/product-support/xpert3-range, accessed on 4 May 2025) and ClayLab ver. 1.0 program were used to process the diffraction data. The identification of mineral phases was based on the PCPDFWIN ver. 1.30 database formalized by JCPDS-ICDD.

The chemical composition of the raw materials was determined by the XRF method using a Philips PW 1404 spectrometer (Philips, Eindhoven, The Netherlands). The excitation source was a double anode X-ray tube (Cr-Au) with a maximum power of 3 kW. Additionally, the total content of Ca, Mg, K, Na, Cl, and SO_4_ ions was determined by ion chromatography using an ICS-5000 chromatograph (DIONEX, Sunnyvale, CA, USA). The content of heavy metals was determined by inductively coupled plasma optical emission spectroscopy (ICP-OES), using an Ultima 2 apparatus from Horiba Jobin-Yvon (Edison, NJ, USA). Dissolved organic carbon (DOC) was determined by the high-temperature combustion method with IR detection. Total dissolved solids (TDS) were determined by the gravimetric method according to PN-EN 15216:2022-03 [57]. Volatile phenols were determined by spectrophotometric method according to PN-ISO 6439:1994 [58]. Loss on ignition (LOI) was determined in accordance with PN-EN 15935:2013-02 [59].

Phase composition of lightweight artificial aggregates were identified using an X-ray diffractometer Miniflex II from Rigaku company (Tokyo, Japan). Filtered copper lamp (CuKα, λ = 0.154 nm), with a voltage of 20 kV, range 2θ = 20–100° and step size 0.02°/3 s was used. Phase composition was determined using the Powder Diffraction File (PDF 2025) developed and issued by the ICDD (The International Center for Diffraction Data).

The obtained lightweight aggregates were subjected to assessment of their physical and mechanical properties, which were determined according to Polish PN standards (Table 2).

Both raw materials and lightweight aggregates were tested for the leachability of chemical substances. The procedure for preparing water extracts from samples was carried out according to the standard [66]. Water extracts for the starting raw materials and aggregate were prepared at a liquid to solid phase ratio of L/S = 10 L/kg (basic test). The leaching liquid was distilled water with pH = 6.3 and electrical conductivity of 0.06 µS/cm. The prepared water extracts were shaken in a laboratory shaker for 24 h and filtered. The analysis of water extracts from raw materials included many determinations. The leachability of heavy metals was determined using ICP-OES methods, while an ion chromatograph was used to analyze selected cations and anions. The concentration of total organic carbon (TOC) in water extracts was determined using a TOC-L analyzer from Shimadzu (Kyoto, Japan). Conductivity and pH of water extracts were determined using the potentiometric method.

Studies on the release of selected substances under flow conditions were also conducted. In this test, distilled water was passed at a speed of 5 m/h through a 10 cm layer of aggregate placed in a vertical filter. In the outflow of this filter, the concentration of selected elements was determined using ion chromatography method, measurements of pH and conductivity. The distilled water used had pH = 7.01 and conductivity at the level of 3.18 µS/cm.

## 3. Results and Discussion

### 3.1. Properties of Raw Materials Used to Produce Lightweight Aggregates

**Figure 2:**
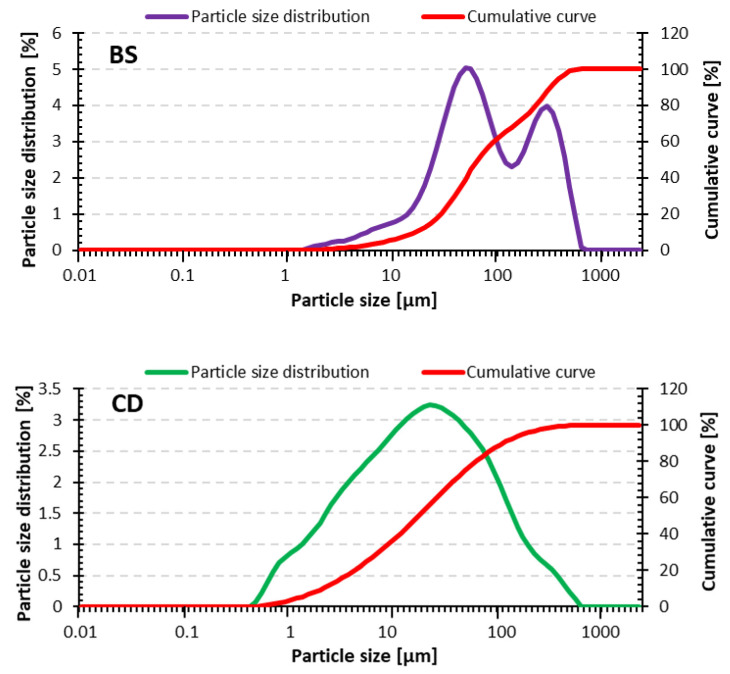

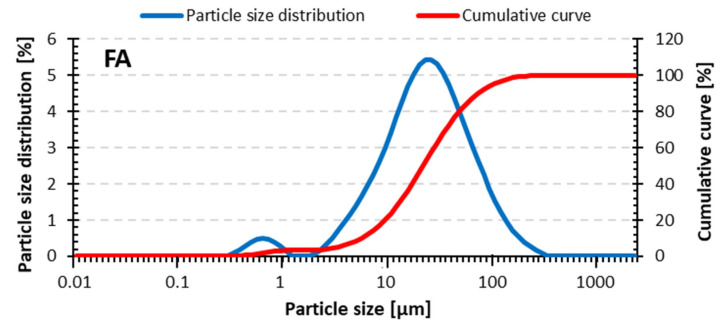
Particle size distribution of raw materials used in the study.

Table 3 shows the grain size parameters of the raw materials used in the study. The D_10_, D_50_, and D_90_ values indicate that the grain size of the bottom sediment was different from that of the other materials, which is a direct result of the origin and preparation method of the materials. Figure 2 shows the grain size distribution of raw materials. A similar grain size distribution was observed for CD and FA. However, the bottom sediment contained significantly larger particles.

The grain size characteristics of the tested fly ash are typical for the MSWI-FA [67,68]. It is dominated by particles below <20 μm. Concrete dust from concrete milling was characterized by a slightly larger share of larger grains. However, the literature also indicates that concrete dust from grinding concrete ceilings can be even finer. Gharpure et al. [69] showed that concrete dust can have over 90% of particles larger than 2.5 μm, which is a significant health problem for construction workers. The grain size distribution of concrete dust is comparable to other mineral waste dusts, such as dolomite dust from aggregate production [70] or granite dust obtained during crushing [71].

**Figure 3:**
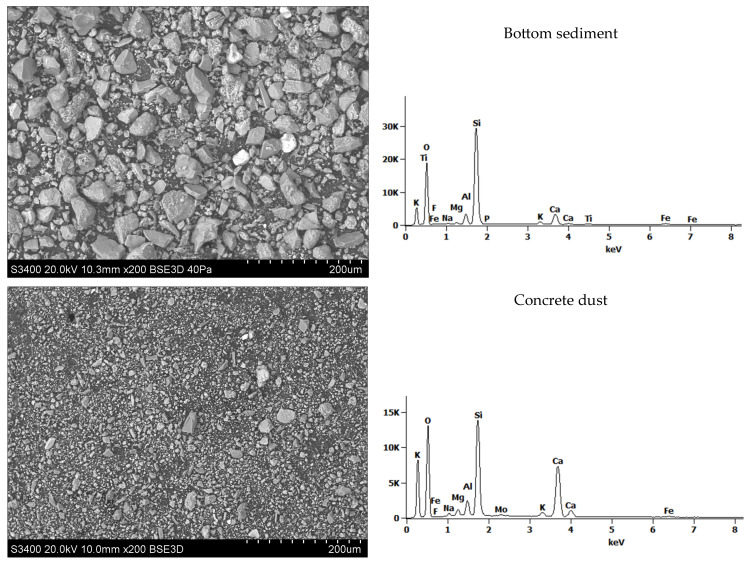

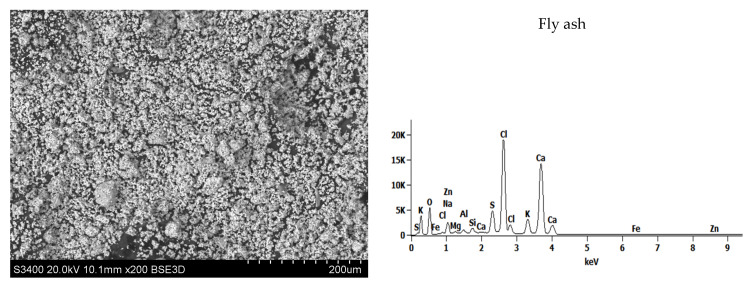
SEM/EDS studies of raw materials.

Scanning electron microscopy (SEM) analysis allowed for a detailed examination of the structure and morphology of raw material grains, which is particularly useful in assessing the suitability of BS, CD, and FA for lightweight aggregate production (Figure 3).

Bottom sediment grains are usually larger and have a more complex structure, often containing organic fragments and minerals. Numerous grains with irregular shapes were observed. Concrete dust grains are characterized by an irregular shape. Their structure can be diverse, with different shapes, from irregular particles to more spherical ones. Fly ash grains are smaller (micrometric) and have a spherical shape. In addition to globular particles, spherical nanometric particles are also observed. They are characterized by a very fine, uniform structure. It can be seen that in fly ash samples irregular grains of variable size with a strongly developed surface showing high porosity of the material with a loose and rough structure prevailed. This can lead to higher water absorption. Chemical analyses in the micro-area (SEM-EDS) showed a different elemental composition. In the bottom sediment and concrete dust, grains with the following chemical compositions dominated, along with grains containing magnesium. The remaining components: iron, sodium and potassium occurred in subordinate amounts. In the case of fly ash, grains containing calcium and chlorine dominated. Potassium had a large elemental share.

X-ray fluorescence (XRF) studies confirmed a clear differentiation of the chemical composition of the analyzed raw materials (Table 4). Each of the materials is characterized by a different elemental profile, which results from their origin and processing. Concrete dust shows a dominance of CaO and SiO_2_, typical for cementitious materials. Bottom sediment contains significant amounts of SiO_2_, Al_2_O_3_, trace elements characteristic of clay materials and mineral detritus. In turn, FA is characterized by the presence of CaO, sulphur and chlorine, which reflects its formation as a result of the combustion of non-homogeneous fractions of municipal waste.

Generally, the chemical and physical characteristics of fly ash depend on the composition of raw municipal waste, operating conditions, incinerator type, and air pollution control system process [34]. The chemical composition shows that the main elements are Si, Al, Fe, Mg, Ca, K, Na, and Cl. In addition, SiO_2_, Al_2_O_3_, CaO, Fe_2_O_3_, Na_2_O, and K_2_O are common oxides found in fly ash [72]. CaO is the most abundant compound in MSWI-FA, while SiO_2_ is the most abundant in MSWI-BA [34]. LOI, expressing the content of unburned carbon in the FA sample, was clearly the highest (37.48%) among the wastes (8.78% for BS; 17.34% for CD), which indicates the presence of unburned waste.

Based on the XRD phase analysis, it was determined that the bottom sediment consisted mainly of quartz SiO_2_, anorthite Ca(Al_2_Si_2_O_8_), and microcline KAlSi_3_O_8_, with a small share of calcite CaCO_3_. In the case of concrete dust, the main phases were quartz SiO_2_, dolomite CaMg(CO_3_)_2_ and calcite CaCO_3_. A similar composition of the debris was observed by Gharpure et al. [69] and Stempkowska & Gawenda [73]. In turn, calcite CaCO_3_, anhydrite CaSO_4_, ettringite 3CaO·Al_2_O_3_·3CaSO_4_·32H_2_O, ghiaraite CaCl_2_·4H_2_O, hibschite Ca_3_Al_2_(SiO_4_)_2_(OH)_4_ and quartz SiO_2_ were diagnosed in the tested fly ash. Both the oxide and phase composition studies confirmed that the raw materials have the appropriate chemical composition and can be used for the production of lightweight aggregate. Their degree of fragmentation is also suitable for carrying out alkaline activation processes [74].

In the context of waste processing, an important aspect is the analysis of the content of hazardous substances. Proper identification and classification of waste in terms of its toxicity are crucial for developing effective strategies for its processing. Waste processing should take into account methods to minimize the risk associated with the release of hazardous substances into the environment, which is essential for sustainable development and the protection of ecosystems [75]. The content of heavy metals and selected organic pollutants in raw materials is presented in Table 5.

Bottom sediment was characterized by the highest content of total organic carbon, which may result from natural organic matter accumulating in the dam reservoir. At the same time, the concentrations of heavy metals and organic pollutants are very low, which indicates a limited environmental risk. Concrete dust shows moderate concentrations of selected heavy metals, such as copper, nickel, barium, as well as moderate the TOC content. The presence of heavy metals results directly from the presence of cement produced from fly ash or slag from the power industry. Due to their chemical properties, these raw materials have the potential for safe and ecological management. Fly ash contained by far the highest concentrations of heavy metals, such as zinc, copper, cadmium, lead, and mercury, as well as an increased level of total PAHs. The content of organic substances did not exceed the limit values established by regulations for hazardous waste [76]. The limit values of harmful substance concentrations, which cause waste to be recognized as hazardous, are: 1.0 mg/kg dm (total PAHs), 6.0 mg/kg dm (BTEX), 1.0 mg/kg dm (PCBs), 30,000.0 mg/kg dm (TOC), and 500.0 mg/kg dm (C_10_–C_40_ hydrocarbons). For the content of heavy metals in waste, there are no established limit values regarding the harmfulness of the waste on the environment.

On the one hand, the content of hazardous substances in the material matrix is important, but the degree of their leaching or release, e.g., during exposure to weather conditions and storage, is more important. The obtained leachability test results were compared with the values that constitute the criterion for accepting waste for storage in landfills and qualifying waste as hazardous waste according to the regulation of the Minister of Economy [76]. The pH of water extracts from raw materials was 8.45, 11.6, and 12.3 for bottom sediment, concrete dust, and fly ash, respectively. In turn, the conductivity reached the level of 224 µS/cm (BS), 522 µS/cm (CD), and 49.4 mS/cm (FA). All eluates of the analyzed samples were characterized by high pH values, which may be related to the presence of oxides. Among the raw materials, fly ash was characterized by the highest leachability, especially chlorides and sulphates. Concentration of chlorides 212 times, sulphates 14 times, and TDS 15 times were higher than the permissible concentrations for inert waste eluates (Table 6). It was found that there is a relationship between the total amount of dissolved solids and electrical conductivity. High leachability is related to the presence of easily soluble chlorides (sylvite KCl, halite NaCl), sulphates (syngenite K_2_Ca(SO_4_)_2_·H_2_O, ettringite, 3CaO·Al_2_O_3_·3CaSO_4_·32H_2_O, gypsum CaSO_4_·H_2_O), oxides (CaO), hydroxides (portlandite Ca(OH)_2_), and carbonates (calcite CaCO_3_) in this type of ash. Leachability of heavy metals was low, because high pH values of the tested waste (pH > 11) resulted in high immobilization of heavy metals in the material. This was confirmed by low concentrations of the tested heavy metals in water extracts. The obtained results confirm the studies of other authors [77,78].

Among the raw materials analyzed, the research showed the potentially hazardous nature of fly ash from incineration waste and the need for its stabilization before potential management. The analysis of the subject indicates that ash constitutes a significant environmental problem due to the presence of hazardous substances. According to the criterion of the regulation [76], ash should be classified as hazardous waste. Their safe and economically justified management is problematic. One of the methods of physicochemical neutralization of fly ash is immobilization (stabilization/solidification). The aim of this process is to transform hazardous waste into a neutral or non-hazardous material so that contaminants occurring in the form of soluble compounds (sulphates, chlorides, heavy metals) do not leach out of it.

### 3.2. The Characteristics of the Obtained Lightweight Aggregates

**Figure 4:**
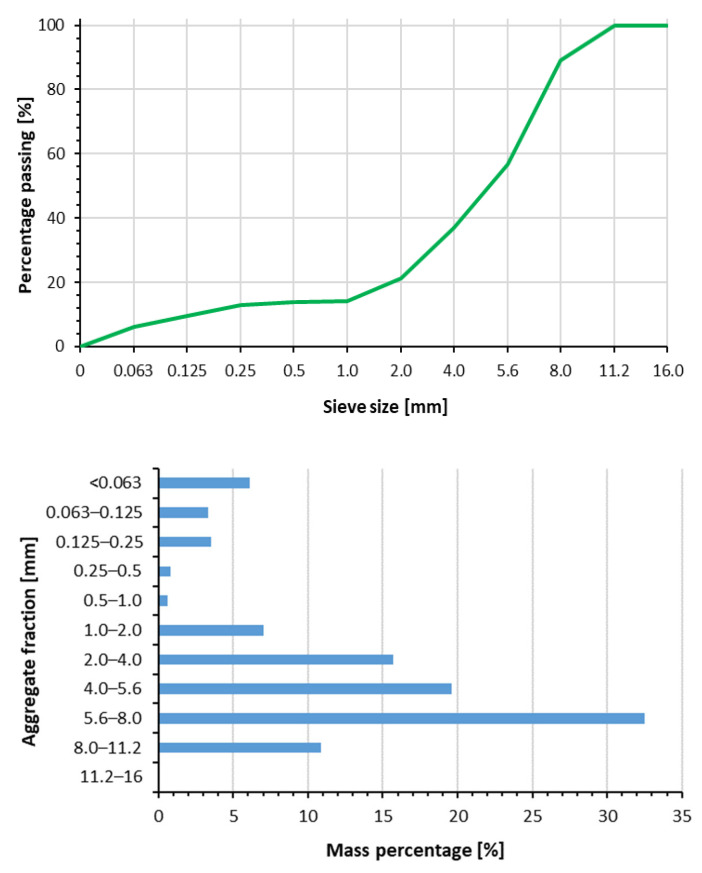
Sieve analysis and division into various size fractions of raw aggregate (aggregate after drying and before the hardening process).

In the process of granulation of a mixture of bottom sediment, concrete dust, and fly ash, aggregate with variable grain size was obtained. The binder during granulation was water. The mixture of raw materials was fed to the granulator continuously in the amount of 50 g/min and was sprayed with water at an intensity of 10 cm^3^/min. The largest grains did not exceed the size of 11.2 mm. Spherical agglomerates with grain size of 5.6–8.0 mm dominated. The share of the <1 mm fraction was at the level of 14.3% (Figure 4).

**Figure 5:**
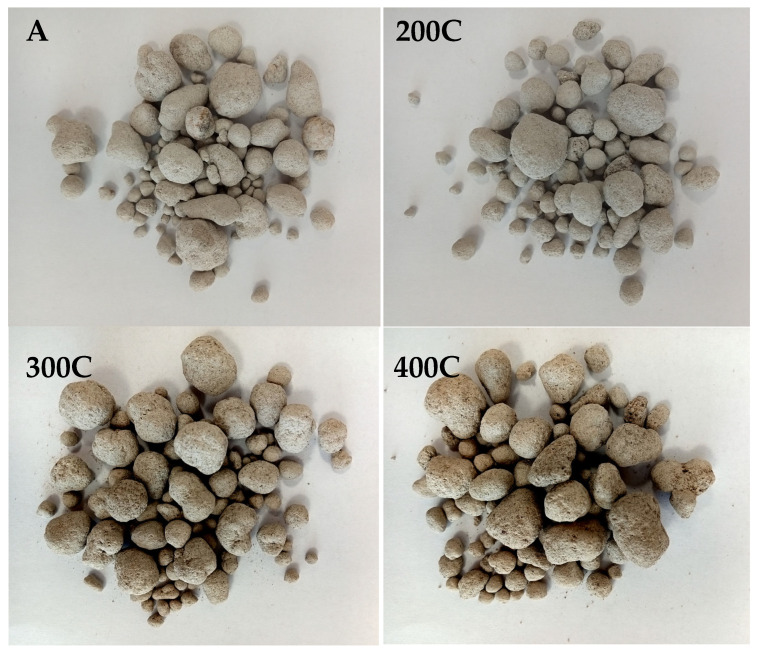
Lightweight artificial aggregate produced in various variants.

The raw aggregate was dried and then subjected to hardening. The experiments resulted in the creation of four types of lightweight aggregate, which were characterized by slightly different properties. Figure 5 shows lightweight aggregates obtained in different process variants.

**Figure 6:**
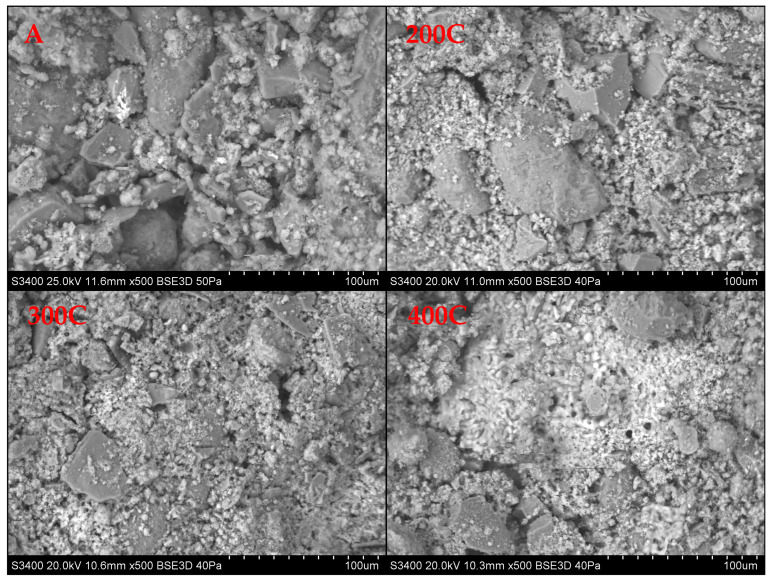
SEM images of aggregates.

The aggregate produced from bottom sediments, concrete dust, and fly ash was characterized by a porous structure, which was documented in SEM images (Figure 6). The tested samples were dominated by round or irregular pores, reaching a diameter of up to several millimeters. It was observed that these pores have a different structure and intensity in the analyzed material. The pores usually do not connect with one another. Analysis of the morphology of empty spaces showed that their presence contributes to a decrease in the specific density and bulk density of the loose material. A compact shell was formed on the surface of the grains subjected to hardening at a temperature of 400 °C. The high porosity of the aggregate is probably related to the presence of fly ash. The low hardening temperature of the aggregate < 400 °C did not lead to the formation of large pores, which is typical for sintering processes at temperatures exceeding 1000 °C. Studies indicate that organic compounds present in raw materials for the production of artificial aggregates are completely destroyed at temperatures above 1000 °C, which results in very high porosity of the final product [48]. Many internal spaces containing silica and oxygen were also detected in the obtained material. These voids are formed as a result of the gas decomposition reaction [79]. This state of affairs affects the mechanical strength, low weight, and thermal and acoustic properties of the material. Elemental analysis at various points shows that the chemical composition of the aggregates is diverse. Studies have shown that the dominant components in the aggregate were calcium, quartz, and chlorine (Table 7).

**Figure 7:**
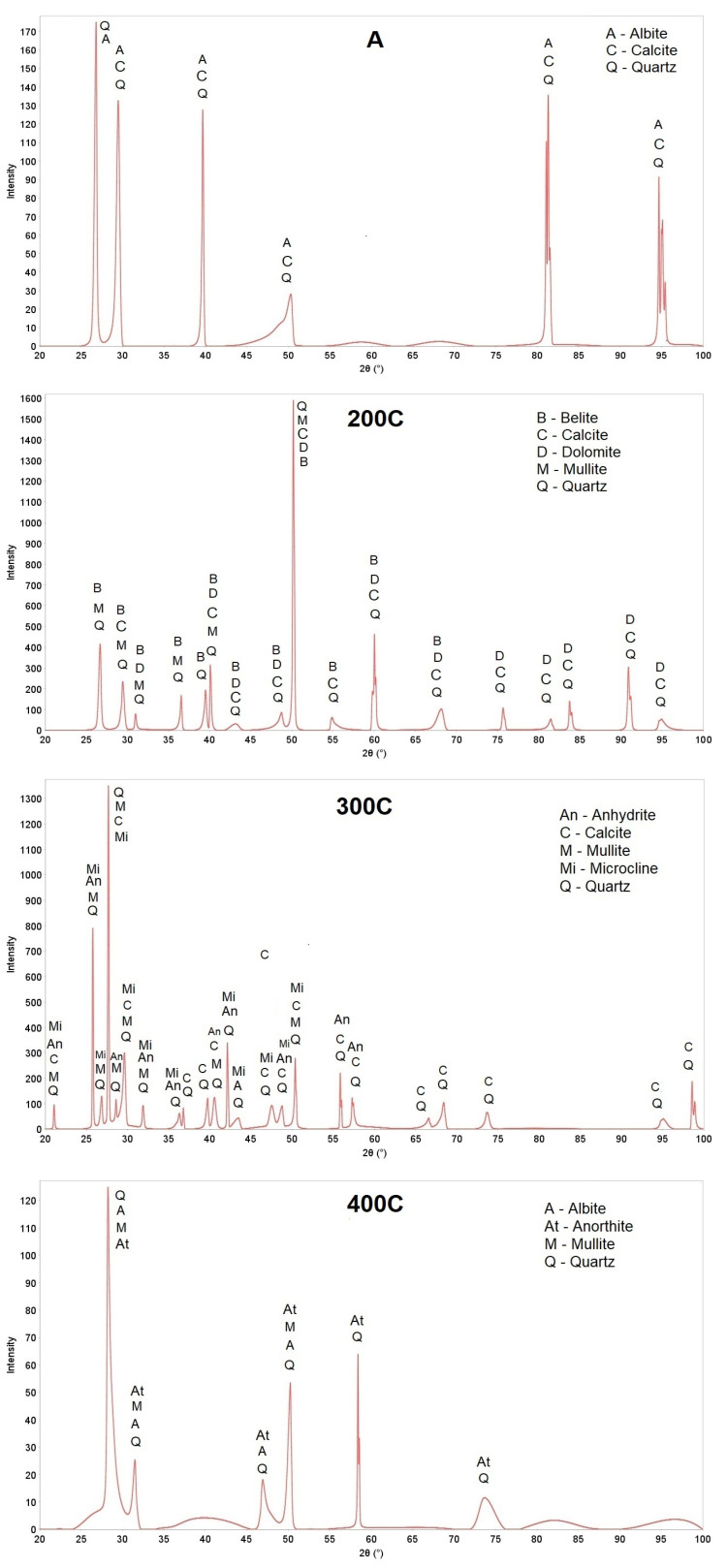
XRD patterns of the aggregates; A—Na_2_O·Al_2_O_3_·6SiO_2_, An—CaSO_4_, At—Ca(Al_2_Si_2_O_8_), B—3Al_2_O_3_·2SiO_2_, C—CaCO_3_, D—CaMg(CO_3_)_2_, M—3Al_2_O_3_·2SiO_2_, Mi—KAlSi_3_O_8_, Q—SiO_2_.

Figure 7 shows the XRD patterns of all tested aggregates. Aggregates have different phase compositions depending on their hardening conditions. First of all, a change in crystalline phases caused by sintering was observed. In the autoclaved aggregate, in addition to quartz and calcite, which occur in waste raw materials, albite was noted. After sintering the three-component mixture with the addition of CaO at a temperature of 200–400 °C, quartz and mullite dominated in each aggregate sample. Calcite, dolomite, and belite were found in the 200C samples. Additionally, anhydrite was found in the 200C sample, and anorthite and belite in the 400C sample. High quartz and calcite contents came from raw materials whose composition was discussed earlier. Due to the low hardening temperature, the rich phase composition characteristic of high-temperature processing was not found [48,80]. At the same time, it should be noted that mineral waste and CaO were used to produce the aggregate in the study and no additional activators, e.g., water glass, were used as in other studies [28].

Based on the analysis of the composition of raw materials and potential processes occurring during the production of artificial aggregate in hydrothermal conditions, it can be assumed that C-S-H phases could have developed in its structure. Crystalline phases (hydrated calcium silicates) are formed in hydrothermal conditions at temperatures above 100 °C and pressures higher than 1 MPa [81].

Selected physical and mechanical properties of aggregates are compared in Table 8. The tests were carried out for samples with grain size of 4–12 mm.

As a result of hardening under autoclaving or sintering conditions, aggregate granules with grains close to spherical were obtained. The number of irregular grains was small. The grain shape index for the obtained aggregates was determined as SI15 for all tested samples, which indicates that less than 15% of the grains show differences in size. These grains were observed to be regular and were therefore classified as spherical according to PN EN 933-4:2008 [64].

The specific density of the obtained aggregates was similar and ranged from 2.61–2.64 g/cm^3^ and was slightly higher than the density of the raw materials. Lightweight porous aggregates, such as, for example, expanded clay aggregate, are characterized by a lower specific density (about 2.5 g/cm^3^) [48]. The grain density of the obtained aggregate grains was less than 1.8 g/cm^3^. The bulk density of the autoclaved aggregate (A) was above 1 g/cm^3^, while that of the low-temperature sintered aggregate was 0.9–0.92 g/cm^3^. For this reason, all types of the obtained aggregate can be classified as lightweight aggregate according to BS EN 13055-2016 [47]. For comparison, artificial aggregates based on fly ash have a lower density. For example, LSA aggregate from fly ash from power plant has a density of 0.796 g/cm^3^ (fraction 4/8 mm) [82]. Aggregate from incineration fly ashes and sediments from the dam reservoir had a density of 0.99 g/cm^3^ (fraction 8/12 mm) [83]. In turn, the lightweight artificial aggregates made with recycled concrete fines and metakaolin, sintered at 350–400 °C, reached a density of 0.78 g/cm^3^ for fractions 4–8 mm and 1.5 g/cm^3^ for fractions 8–16 mm [28]. Another type of aggregate obtained from concrete dust and ladle slag, hardened at 60 °C, had a density of 0.965 g/cm^3^ [29]. The QTA aggregate from fly ash, cement, and waste quartz autoclaved at a temperature of 195 °C and a pressure of 1.38 MPa had a bulk density varied from 0.998 to 1.087 g/cm^3^, and the apparent density ranged from 1.553 g/cm^3^ to 1.818 g/cm^3^ [84]. The bulk density of materials is closely related to their structure and porosity. A higher value of the bulk density may suggest that the material is more compact. In addition, a higher value of this parameter may also indicate higher compressive strength [48].

In the tested aggregates, the closed porosity ranged from 11.2 to 16.8%, which means that a significant portion of pores is accessible to liquids or gases. The open porosity is slightly higher and amounts to 16.8–22.6%, which indicates a large number of open spaces in the grain structure. The total porosity in the form of the sum of both of these values ranges from 31.5 to 36.0%, depending on the aggregate hardening conditions. Compared to other lightweight aggregates, this parameter is similar to expanded clay aggregate, the porosity of which ranges from 20 to 50% [48]. Stempkowska & Gawenda [73] obtained aggregate from concrete dust, clay, and PET bottles with a porosity of 30%. High porosity minimizes water absorption and additionally provides infiltration properties, i.e., the ability of gravitational water flow and high water vapor diffusivity [48].

The studies showed the water absorption of the aggregate in the range of 9.63–13.43% depending on the hardening conditions. The water adsorption of the obtained material is closely related to the number and size of pores. The studies showed that the pores are not fully saturated with water. The pores formed during the hardening process in this study turned out to be closed pores, which resulted in reduced water permeability. It is similar to the water absorption for aggregates from concrete dust and ladle slag (10.7–13.00%) [29] or lightweight aggregates obtained from fly ash using microwave radiation (11–14%) [51]. Leca aggregate has a water absorption of 30% [85]. Other lightweight aggregates from waste, such as KRC aggregates, show water absorption of 33–35% [28], while another autoclaved quartz tailing lightweight aggregate (QTA) showed water absorption of 13.77–21.93% [84]. According to Hao et al. [49], water absorption by lightweight aggregate is dependent on influencing factors, including the type of materials used, the type of hardening, and the type of binder used. The high-temperature sintering method of 1000 °C showed that as the temperature increases, the water absorption of lightweight aggregate decreases because it contains closed pores.

The results of the frost resistance tests indicate that the aggregate showed the lowest mass loss for the autoclaved sample (4.45%). Sintering at low temperatures of 200 °C and 300 °C worsened frost resistance but did not exceed 5%. However, for aggregate 400C the mass loss increased to 8.32%. Observations of aggregate grains showed damage to the outer layer. This phenomenon can be attributed to the granulation process, in which the binder bond was weaker with a thicker layer of material. Similar observations were shown by Gosk et al. [28].

The tests carried out showed the aggregate compressive strength at a very diverse level. The autoclaved sample (A) obtained a value of 0.684 MPa. At low sintering temperatures, comparable results were obtained, respectively 0.063 MPa (200C) and 0.609 MPa (300C). The use of a temperature of 400 °C for sintering caused a drastic decrease in the compressive strength of aggregate, to the level of 0.27 MPa, which may disqualify its usefulness. Such a decrease can be attributed to possible calcination and decomposition of carbonates, which leads to a weakening of the material structure. These results emphasize the importance of the appropriate selection of the sintering temperature in the process of aggregate production from bottom sediments, concrete dust, and fly ash, in order to ensure optimal mechanical properties. Higher compressive strength is possessed by, among others, lightweight expanded aggregates from smectite clay, palygorskite-rich sediment and phosphate sludge (0.86 MPa) [86], and KRC aggregate 4–8 mm (1.17–1.23 MPa) [28]. Hao et al. [49] showed that aggregates with the addition of water glass or cement can be characterized by high strength parameters. Artificial aggregates can achieve strengths of even over 10 MPa, e.g., phosphogypsum-based cold-bonded aggregates [87].

The properties of lightweight aggregates largely determine their later application. According to Franus [48], the lightweight aggregate obtained in this study with a bulk density (0.9–1.02 g/cm^3^), water absorption (9.63–13.43% by weight) and compressive strength (0.609–0.684 MPa) can be used in lightweight non-structural concretes, lightweight mortars, geotechnics, horticulture, landscaping, and thermal and acoustic insulation. In terms of use, the leachability of harmful components of the finished aggregate is also important. This applies primarily to the use of aggregate in geotechnics or horticulture, when the material may have direct contact with the natural environment.

### 3.3. Ecological Aspect of Lightweight Artificial Aggregates

The assessment of the ecological properties of the produced lightweight aggregate types was carried out based on a comparison of the composition of the water extract with the permissible limits specified for waste [76] and the limit values for the water extract, treated as wastewater [88]. The chemical composition of the water extract characterizes the quantity and quality of water-soluble substances contained in the material subjected to testing. Table 9 and Table 10 present the results of the leachability of selected chemical substances from the matrix of aggregates.

The pH of water extracts from aggregates was 10.38 (A), 10.88 (200C), 10.97 (300C), and 10.81 (400C). In turn, the conductivity reached the level of 10.06 mS/cm, 10.43 mS/cm, 10.25 mS/cm, and 11.44 mS/cm for A, 200C, 300C, and 400C sample, respectively. The studies have shown that heavy metals have been immobilized in the aggregate structure, and their leachability is negligible, so the aggregates do not pose a threat to the aquatic environment. Similarly, this applies to organic substances, including hydrocarbons, which were partially removed under hardening conditions. The studies clearly showed the above-standard leachability of chlorides, sulphates, and TDS from the aggregate. Comparison with the values in Table 6 shows that the obtained aggregates, especially those hardened at 200–400 °C, cannot be considered as neutral materials.

The aggregates produced, hardened at low temperatures, may pose a significant threat to the environment in the event of direct exposure to atmospheric conditions, which leads to the leaching of substances by atmospheric precipitation. They can only be used as aggregate for the production of lightweight concrete, which results in their closure in the concrete structure, limiting contact with external factors. A comparison of the eluate test results with the limit values specified in the regulation [88] indicates that all the analyzed indicators meet the requirements, except for the content of chlorides and sulphates, which exceed the permissible values (Table 11).

The quality parameters of the water extract from aggregates clearly result from the use of fly ash from waste incineration. Therefore, the use of a significant amount of this type of fly ash in the aggregate production mixture is problematic from the point of view of obtaining an ecologically safe product. For comparison, KRC aggregate produced from concrete dust, also at comparable sintering at low temperatures (400 °C), did not contain excessive amounts of hazardous substances [28].

**Figure 8:**
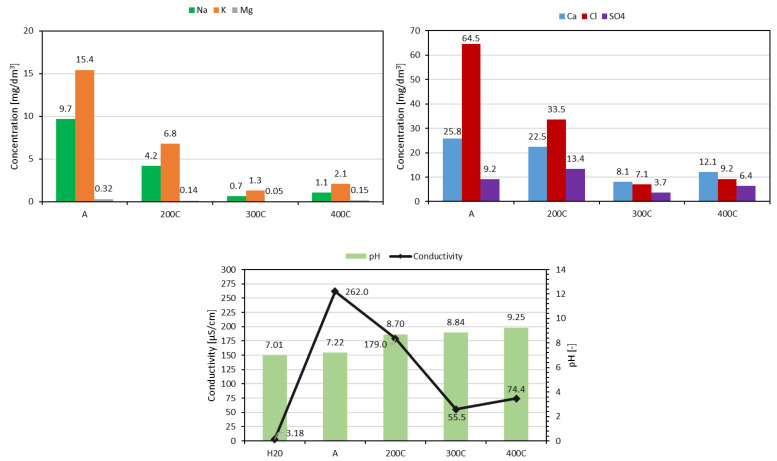
Leachability of selected components from aggregate under flow conditions.

Leachability tests in flow conditions were aimed at determining the potential emission of selected components from lightweight aggregate, primarily chlorides and sulphates, during filtration—for example, when the aggregate is used as a drainage material in horticulture or on green roofs. The test results showed that the water runoff after filtration through autoclaved aggregate (A) was characterized by the lowest pH and the highest values of electrical conductivity and content of all analyzed components, except sulphates. On the other hand, filtrates from aggregates hardened at 200 °C, 300 °C, and 400 °C were characterized by lower salinity but higher pH (Figure 8).

On the one hand, the total leachability tests showed that lightweight aggregates subjected to thermal hardening at a temperature of 200–400 °C do not provide full immobilization of chlorides and sulphates. On the other hand, they also showed that water flow in filtration conditions causes less leaching of these components compared to autoclaved aggregate. Therefore, from an ecological point of view, the use of individual types of aggregates may be different and dependent on the specifics of the project and environmental conditions. In this context, the use of lightweight aggregates for the production of lightweight concrete becomes a real and justified possibility, provided that appropriate protection against the adverse effects of atmospheric factors is implemented. When using aggregate for concrete, the presence of chlorides and sulphates must also be taken into account due to chloride and sulphate corrosion in concrete [89].

## 4. Conclusions

In these studies, the possibilities of using bottom sediment, concrete dust, and municipal solid waste incineration fly ash for lightweight artificial aggregates production in low-temperature sintering and autoclaving process were evaluated. The results reveal that the application of fly ash together with bottom sediment and concrete dust in aggregates are feasible. However, there are some environmental limitations. Studies of aggregates produced in various variants have shown that aggregates subjected to thermal hardening at temperatures in the range of 200–400 °C do not ensure full immobilization of pollutants such as chlorides and sulphates. On the other hand, in this aspect, hardening by sintering proved to be more effective compared to aggregates obtained in the autoclaving process. In turn, slightly different observations were found in the analysis of strength parameters of the tested aggregates. In this case, the autoclaving process proved to be more effective. Generally, the autoclaving process produced aggregates with strength comparable to the widely used expanded clay. The results of the conducted studies suggest that the use of this type of aggregate for the production of lightweight concrete is becoming the most realistic and justified possibility (assuming appropriate protection against the adverse effects of atmospheric factors). Moreover, the developed lightweight aggregate production process has significantly lower energy consumption compared to conventional lightweight aggregate production methods, which require high-temperature sintering, usually 1000–1200 °C. The use of autoclaving and sintering in the range of 200–400 °C makes it possible to reduce the hardening temperature range by as much as 65–75%, resulting in a more sustainable production process. Nevertheless, in the aspect of implementing pro-ecological production technologies, the use of individual types of aggregates may be different and dependent on the specificity of the project and environmental conditions.

## 5. Patents

As a result of the research presented in this publication, two technological solutions were developed and submitted for patent protection [55,56].

## Figures and Tables

**Table 1:** Variants of aggregates prepared for testing.

Sample	Granulation	Drying (Temperature, Time)	Hardening (Type, Temperature, Time)
A	rotating dish granulator	shelf dryer 60 °C 4 h	Autoclave, 180 °C, 1 h,
200C			Muffle oven, 200 °C, 1 h
300C			Muffle oven, 300 °C, 1 h
400C			Muffle oven, 400 °C, 1h

**Table 2:** Methodology of research concerning the physical and mechanical characteristics.

Parameter	Standard
Grain density	PN-EN 1097-3:2000 [60]
Bulk density	PN-EN 1097-3:2000 [60]
Loose bulk density	PN-EN 1097-3:2000 [60]
Porosity	PN-EN 1097-3:2000 [60]
Compressive strength	PN-EN 1097-11:2013 [61]
Frost resistance	PN-EN 1367-1:2007 [62]
Water absorption	PN-EN 1097-6:2022-07 [63]
Grain shape index	PN EN 933-4:2008 [64]
Grain composition	PN-EN 933-1:2012 [65]

**Table 3:** Particle size analysis of raw materials.

Raw Material	D_10_ [μm]	D_50_ [μm]	D_90_ [μm]
Bottom sediment	13.8	57.6	312.0
Concrete dust	2.21	17.7	100.0
Fly ash	4.1	18.1	58.7

**Table 4:** XRF oxide analysis of raw materials.

Component, wt. %	BS	CD	FA
Na_2_O	n.o.	n.o.	1.71
MgO	1.15	1.59	0.19
Al_2_O_3_	9.36	2.85	0.53
SiO_2_	65.53	34.40	1.48
P_2_O_5_	0.32	n.o.	n.o.
SO_3_	0.46	0.85	6.99
Cl	n.o.	0.03	15.59
K_2_O	2.82	1.08	3.89
CaO	5.31	39.39	29.82
TiO_2_	0.80	0.19	0.30
MnO	0.14	0.06	0.05
Fe_2_O_3_	5.15	2.13	0.58
CuO	n.o.	n.o.	0.04
ZnO	0.02	0.04	1.04
Br_2_O_3_	0.04	n.o.	0.11
SrO	0.02	0.04	0.04
ZrO_2_	0.06	n.o.	n.o.
BaO	0.02	0.03	0.02
PbO	n.o.	n.o.	0.13

n.o.—not observed.

**Table 5:** Content of heavy metals and other hazardous substances in raw materials [mg/kg per dry mass].

Component, mg/kg dm	BS	CD	FA
Total PAHs ^1^	<0.01	0.085	0.3
BTEX ^2^	<0.1	<0.1	<0.1
PCBs ^3^	<0.001	<0.001	<0.001
TOC ^4^	16,800	9936	7663
C_10_–C_40_ hydrocarbons	19	22	11
As	<5	<5	<5
Ba	17.7	31.9	108
Cd	<0.5	<0.5	48.5
Cr	8.41	6.72	14.4
Cu	<5	109	169
Hg	<0.25	<0.25	5.42
Mo	<5	<5	<5
Ni	<5	63.2	6.61
Pb	<5	<5	677
Sb	<5	<5	202
Se	<5	<5	<5
Zn	12.6	16.1	5862

^1^ PAHs—Polycyclic aromatic hydrocarbons; ^2^ BTEX—the concentration of Benzene, Toluene, Ethylbenzene, and Xylene; ^3^ PCBs—polychlorinated biphenyls; ^4^ TOC—Total Organic Carbon.

**Table 6:** Comparison of the leaching values of selected components with the values allowing for storage of a given type of raw material (in mg/kg per dry mass).

Component, mg/kg dm	Raw Material			Limit Values [76]		
	BS	CD	FA	Inert Waste	Waste Other than Inert and Hazardous	Hazardous Waste
Ca	360.6	584.3	63,374.0	not limited	not limited	not limited
Mg	40.52	7.3	8.65	not limited	not limited	not limited
K	27.25	461.1	41,598.9	not limited	not limited	not limited
Na	11.73	151.6	27,773.7	not limited	not limited	not limited
Fl	<1	6.3	39	10.0	150.0	500.0
Cl	31	890	170,000	800.0	15,000.0	25,000.0
SO_4_	300	300	14,000	1000.0	20,000.0	50,000.0
As	<0.1	<0.1	<0.1	0.5	2.0	25
Ba	0.33	2.28	19.3	20.0	100.0	300.0
Cd	<0.01	<0.01	<0.01	0.04	1.0	5.0
Cr	<0.05	0.069	<0.05	0.5	10.0	70.0
Cu	<0.05	<0.05	<0.05	2.0	50.0	100.0
Hg	<0.001	<0.001	<0.001	0.01	0.2	2.0
Mo	<0.2	<0.2	0.81	0.5	10.0	30.0
Ni	<0.1	<0.1	<0.1	0.4	10.0	40.0
Pb	<0.1	<0.1	<0.1	0.5	10.0	50.0
Sb	<0.01	<0.01	<0.01	0.06	0.7	5.0
Se	<0.1	<0.1	<0.1	0.1	0.5	7.0
Zn	<0.2	<0.2	2.92	4.0	50.0	200.0
TDS	1900	39,200	322,000	4000.0	60,000.0	100,000.0
DOC	250	169	443	500.0	800	1000.0
Volatile Phenols	<0.25	0.78	0.47	1.0	not limited	not limited

TDS—Total Dissolved Solids, DOC—Dissolved Organic Carbon.

**Table 7:** Atomic percentage [%] of select elements at aggregates by EDS method.

Sample	O	Ca	Si	Na	Mg	Al	K	Fe	Cl	S	Zn
A	33.9	37.7	13.7	0.5	0.7	1.6	1.7	0.9	5.1	3.2	0.6
200C	34.4	33.9	15.2	1.5	0.6	1.8	2.0	1.0	6.8	2.4	0.9
300C	33.0	34.2	14.1	0.8	0.5	1.6	3.0	1.3	9.0	2.3	0.6
400C	31.4	36.7	11.45	1.8	0.5	1.5	3.3	1.3	9.6	2.0	0.4

**Table 8:** Physical and mechanical properties of the produced lightweight aggregate.

Parameter	Unit	A	200C	300C	400C
Grain density	g/cm^3^	1.74 ± 0.05	1.69 ± 0.08	1.79 ± 0.07	1.76 ± 0.03
Bulk density	g/cm^3^	1.02 ± 0.03	0.92 ± 0.02	0.92 ± 0.03	0.90 ± 0.03
Specific density	g/cm^3^	2.62 ± 0.06	2.63 ± 0.05	2.61 ± 0.05	2.64 ± 0.07
Total porosity	%	33.6 ± 2.03	36.0 ± 1.49	31.5 ± 0.89	33.4 ± 1.21
Open-pore porosity	%	16.8 ± 2.89	22.6 ± 1.60	20.3 ± 2.31	19.9 ± 6.48
Compressive strength	MPa	0.684 ± 0.23	0.663 ± 0.26	0.609 ± 0.18	0.270 ± 0.10
Frost resistance	%	4.45	4.60	4.82	8.32
Water absorption	%	9.63 ± 1.92	13.43 ± 1.43	11.35 ± 4.44	11.30 ± 3.80
Grain shape index	%	9.1	10.2	8.5	12.0

**Table 9:** Content of heavy metals and other hazardous substances in aggregates [mg/kg dry mass].

Component, mg/kg dm	A	200C	300C	400C
Total PAHs	<0.01	0.02	0.02	<0.01
BTEX	<0.01	<0.01	<0.01	<0.01
PCBs	<0.001	<0.001	<0.001	<0.001
TOC	6460.0	6844.0	6620.0	6543.0
C_10_–C_40_ hydrocarbons	8.5	6.2	4.25	2.64
As	<5	<5	<5	<5
Ba	39.2	39.1	39.4	39.2
Cd	9.2	9.3	9.4	9.4
Cr	8.4	8.5	8.5	8.4
Cu	73.15	72.9	73.1	73.30
Hg	1.1	1.0	1.1	1.1
Mo	<5	<5	<5	<5
Ni	26.4	26.6	25.9	25.9
Pb	130.6	130.8	1307	130.5
Sb	40.6	40.8	40.5	40.7
Se	<5	<5	<5	<5
Zn	1116.8	1116.6	1117.1	1116.8

**Table 10:** Comparison of the leaching values of selected components with the values allowing for storage of a given type of aggregates (in mg/kg dm).

Component, mg/kg dm	A	200C	300C	400C
Ca	14,552.0	14,084.3	13,184.2	15,988.7
Mg	43.0	41.7	49.4	50.1
K	6771.6	7339.4	7106.6	7871.5
Na	4770.1	5130.0	4953.3	5551.1
Fl	4.4	5.2	6.3	8.1
Cl	31,721.1	35,344.2	35,237.1	39,314.8
SO4	11,816.4	9080.6	6932.9	12,050.9
As	<0.01	<0.01	<0.01	<0.01
Ba	3.2	3.8	4.12	4.55
Cd	<0.01	<0.01	<0.01	<0.01
Cr	<0.05	<0.05	<0.05	<0.05
Cu	<0.05	<0.05	<0.05	<0.05
Hg	<0.001	<0.001	<0.001	<0.001
Mo	0.26	0.24	0.22	0.30
Ni	<0.01	<0.01	<0.01	<0.01
Pb	<0.01	<0.01	<0.01	<0.01
Sb	<0.001	<0.001	<0.001	<0.001
Se	<0.1	<0.1	<0.1	<0.1
Zn	0.48	0.44	0.61	0.68
TDS	45,482.6	51,247.5	68,592.4	71,258.5
DOC	84.6	86.3	72.8	186.5
Volatile Phenols	0.3	0.34	0.28	0.25

**Table 11:** Selected components in water extracts from aggregates.

Component, mg/dm^3^	A	200C	300C	400C	Limit Values [88]
Cl	3172.1	3534.4	3523.7	3931.5	1000.0
SO_4_	1181.6	908.1	693.3	1205.1	500.0

## Data Availability

The original contributions presented in this study are included in the article. Further inquiries can be directed to the corresponding author.
